# Discussion to: Identifying lung cancer disparities among Asian Americans: A novel analytic approach

**DOI:** 10.1016/j.xjon.2024.05.001

**Published:** 2024-05-15

See Article page 153.

Presenter: Ms Yunna Gu

**Dr Anthony W. Kim***(Los Angeles, Calif)*. I would like to thank the American Association for Thoracic Surgery for the privilege of discussing this paper. I would also like to thank Miss Gu, Dr Lazar, and coauthors for providing me with both the manuscript and the presentation in a timely manner, well before our meeting. Furthermore, I appreciate the authors taking extra time out of their busy schedules to review their methodology in greater detail prior to their presentation today. As their title indicates, a novelty of their statistical analysis to evaluate a small but growing number of Asian Americans with lung cancer, and the disparities in the care associated with this disease should prove to be valuable information in a previously sparsely occupied space. In broad strokes, the authors compare Asian Americans with lung cancer as a whole, and as ethnic subgroups to determine underrepresentation and overrepresentation in both clinical stage and vital status. From the cross-tabulation analysis, they provide insight into socioeconomic differences across these cohorts, perhaps dispelling some preconceived notions about Asian Americans. From their χ^2^ and columnar Z-score analysis, they ultimately conclude that Asian Americans are more likely to be diagnosed with advanced stages of lung cancer yet have a higher associated survival rate compared with White Non-Hispanic, Black, and/or Hispanic patients. Their effort represents a terrific proof of concept approach that may be applicable to other thoracic surgeon scientists pursuing similar lines of work. That said, I do have some clinical questions. You hinted at this, but in your manuscript, you have a dense amount of socioeconomic information in your cross-tabulation results that you couldn not present in the interest of time. So for the benefit of the general audience, can you distill how underrepresentation factors such as the first quartile of income for all Asian American subgroups and overrepresentation of some Asian American subgroups such an East Asians in the fourth quartile of education, as well as other findings, such as Southeast Asian Americans and South Asian Americans being overrepresented in the private insurance groups play into your final results?

**Figure:**
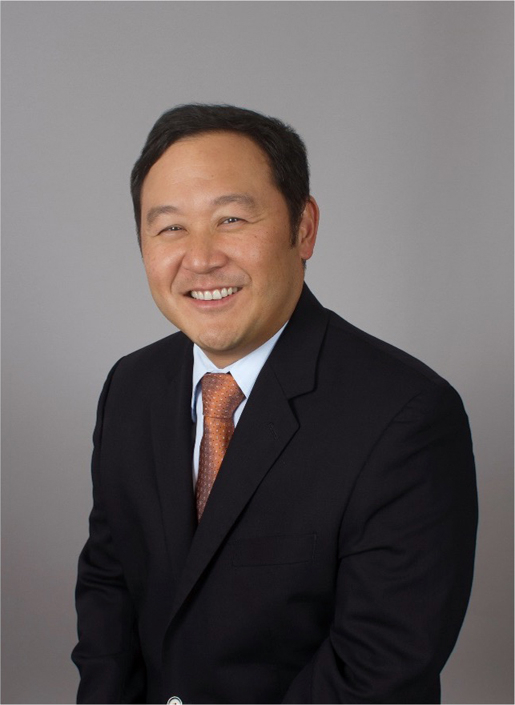

**Ms Yunna Gu** (*Washington, DC*). Thank you, Dr Kim. Thank you for your questions. To answer this question, I must bring up the Model Minority Myth, which surfaced in 1960s, describing the success of Japanese and Chinese Americans despite discrimination. When we generalize a group and label them as the model minority, we run into the risk of masking unique subgroup differences that are identifiable and assume similar disease risk as the White Non-Hispanic majority. Our data show all Asian American subgroups were underrepresented in the first median income quartile and overrepresented in the fourth median income quartile relative to White Non-Hispanic, whereas Black and Hispanic subgroups showed the opposite. East Asian and South Asian were also overrepresented in the first quartile of noncompletion of high school degree, which is <6.3% of noncompletion rate of high school degree compared to White Non-Hispanics. All Asian American subgroups were overrepresented in the first quartile of noncompletion of high school degree relative to Black.

**Figure:**
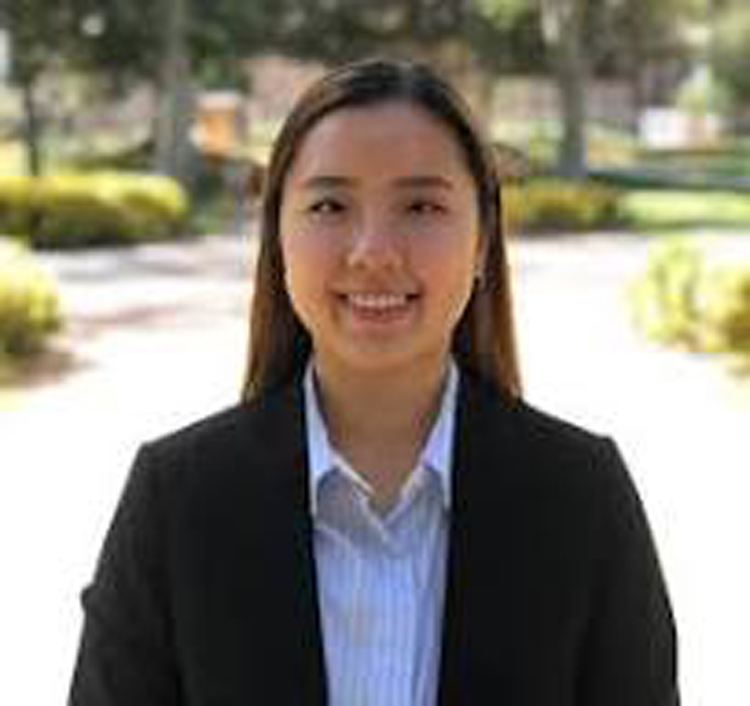

Looking at these data, one can come to the false assumption of the model minority. But when we look at data more closely, variation between the subgroups exist, and Asian Americans might not be doing as well as we assumed. All the Asian American subgroups were also overrepresented in the fourth quartile of noncompletion of high school degree, which is ≥17.6% of noncompletion rate compared to White Non-Hispanic. This is interesting because we see both overrepresentation in first and fourth quartile of noncompletion of high school degree in Asian American subgroups. Many Asian Americans abroad complete their education prior to immigrating to the United States, so when we talk about noncompletion of high school degree, we tend to think about US-born Asian Americans. So long-winded answer to your question. Despite the model minority myth and presumed higher socioeconomic status, all Asian American subgroups were more likely to be not insured than White Non-Hispanic. And on top of that, they were underrepresented as Medicare primary payers, and overrepresented as Medicaid primary payers, as were Black and Hispanic. To summarize, we cannot solely examine Asian American as one group, and there must be factors other than socioeconomic status that are influencing the health disparity that we see today.

**Dr Kim**. Great, thank you. Two more quick questions. The genetic predispositions to lung cancer that Asians carry notwithstanding, do you have any historical information or other data that provide context regarding the tobacco burden and/or participation in lung cancer screening programs? Or for that matter, any intervention that should be enacted in these domains to perhaps to prevent or identify earlier cancers in Asian Americans, respectively?

**Ms Gu**. Thank you for the question. Smoking is an accepted social behavior in many Asian countries. And sometimes, it is necessary for social and business interactions. So, it is highly prevalent, especially in Asian men. The idea of collectivism, which is a philosophy of Confucianism, may foster group behaviors like smoking. It was found that Asian American men were more likely to be current smokers if they had a current smoker father or brother, and/or current smoker friends. In terms of screening, lung cancer screening has poorly penetrated Asian Americans as they have lower rates of cancer screening than Whites Non-Hispanic. Asian Americans are actually the only racial group where cancer screening disparity is not well explained by socioeconomic factors.

Of course, culture plays a big role. For example, Confucianism also views illnesses as one's fate, so preventative health care behaviors are not common or as accepted in Asian American cultures. So, to prevent or identify earlier cancers in Asian Americans, the first step is to recognize that these underlying cultural differences exist. We need to have more relevant programs that look at social and culture norms that relate to cancer risk behaviors and provide culturally sensitive screening education for our patients. And also, something else I mentioned earlier is the fact that a lot of surveys are conducted in English, and more than one-third of Asian Americans speak English less than “very well”. So, one point to make is we should have health education material and surveys conducted in ethnic-specific languages.

**Dr Kim**. Great. Last question. I will try to be brief. Like many ethnic groups in the United States, there is a tendency to cluster in accordance with geography. Were you able to consider a different type of analysis to account for this phenomenon? If not or if so, do you believe that this type of aggregation contributes, or has the potential to help with improved education around lung cancer or other diseases that are more prevalent or more impactful in Asian American communities, or any community for that matter? I imagine that the regions including and surrounding Washington, DC, to some extent informed your research group of the value of pursuing a study such as yours. So how could this study inform other communities in which minorities are clustered?

**Ms Gu**. I mentioned a lot of times during my talk that aggregating Asian American as one group is not sufficient to study subgroup health disparities. Even though some can still consider what we did in our study as aggregating Asian Americans, to ensure statistical significance, we had to aggregate them into 3 subgroups based on geographic origins. So, this is the first step in disaggregating Asian American subethnicities. In the future, we hope to work with medical centers in densely Asian American populated regions to be able to study these individual ethnicities. And on top of that, if we were able to have smoking status and language preferences included in the database or in a study or survey, we would be able to assess the interplay of those factors as well.

**Dr Kim**. Okay. I just wanted to congratulate you guys on your innovative work, and in particular, you. As a third-year student, I applaud you for being a forceful and fervent champion for Asian Americans with lung cancer so early in your career.

**Ms Gu**. Thank you so much. Thank you.

## Conflict of Interest Statement

The authors reported no conflicts of interest.

The *Journal* policy requires editors and reviewers to disclose conflicts of interest and to decline handling manuscripts for which they may have a conflict of interest. The editors and reviewers of this article have no conflicts of interest.

